# Osteoprotective Activity and Metabolite Fingerprint via UPLC/MS and GC/MS of *Lepidium sativum* in Ovariectomized Rats

**DOI:** 10.3390/nu12072075

**Published:** 2020-07-13

**Authors:** Hossam M. Abdallah, Mohamed A. Farag, Mardi M. Algandaby, Mohammed Z. Nasrullah, Ashraf B. Abdel-Naim, Basma G. Eid, Martin K. Safo, Abdulrahman E. Koshak, Azizah M. Malebari

**Affiliations:** 1 Department of Natural Products and Alternative Medicine, Faculty of Pharmacy, King Abdulaziz University, Jeddah 21589, Saudi Arabia; aekoshak@kau.edu.sa; 2 Department of Pharmacognosy, Faculty of Pharmacy, Cairo University, Cairo 11562, Egypt; mohamed.farag@pharma.cu.edu.eg; 3 Department of Chemistry, School of Sciences & Engineering, The American University in Cairo, New Cairo 11835, Egypt; 4 Department of Biological Sciences, Faculty of Science, King Abdulaziz University, Jeddah 21589, Saudi Arabia; malgandaby@kau.edu.sa; 5 Department of Pharmacology and Toxicology, Faculty of Pharmacy, King Abdulaziz University, Jeddah 21589, Saudi Arabia; mnasrullah@kau.edu.sa (M.Z.N.); aaabdulalrahman1@kau.edu.sa (A.B.A.-N.); beid@kau.edu.sa (B.G.E.); 6 Department of Medicinal Chemistry, Institute for Structural Biology, Drug Discovery and Development, School of Pharmacy, Virginia Commonwealth University, Richmond, VA 23298, USA; msafo@vcu.edu; 7 Department of Pharmaceutical Chemistry, College of Pharmacy, King Abdulaziz University, Jeddah 21589, Saudi Arabia; amelibary@kau.edu.sa

**Keywords:** osteoprotection, *Lepidium sativum*, anti-oxidation, phenolics, ovariectomized rats, LC-MS fingerprint

## Abstract

*Lepidium sativum* seeds are used traditionally to accelerate healing of bone fracture in addition to its culinary uses. This study aimed to characterize the osteoprotective effect of *L. sativum* in an ovariectomized rat model at two dose levels (50 and 100 mg/kg) using 17β-estradiol as a positive reference standard. Moreover, a complete metabolite profile of *L. sativum* via UHPLC/PDA/ESI–MS, as well as headspace solid-phase microextraction (SPME)-GC/MS is presented. Results revealed that *L. sativum* extract exhibited significant anti-osteoporotic actions as evidenced by mitigating the decrease in relative bone weight concurrent with improved longitudinal and perpendicular femur compression strength. Further, the extract enhanced the serum bone formation biomarkers lactate dehydrogenase (LDH) activity and osteocalcin levels. The extract also inhibited exhaustion of superoxide dismutase (SOD) as well as glutathione peroxidase (GPx) activities and accumulation of lipid peroxides in bone tissues. This is in addition to ameliorating the rise in the markers of bone resorption carboxyterminal telopeptide, type I (CTXI) and tartrate-resistant acid phosphatase (TRAP) and modulating receptor activator of nuclear factor kappa-Β ligand (*RANKL*)/ osteoprotegerin (*OPG*) expression. Metabolite characterization suggests that glucosinolates, lignans, coumarins, phenolic acids, and alkaloids mediate these anti-osteoporotic effects in a synergistic manner.

## 1. Introduction

Osteoporosis is a bone disorder, which is a consequence of imbalance in the formation and resorption of bone tissues in favor of bone loss, with elevated fracture risk. In the last two decades, osteoporosis has become a major health problem affecting a huge worldwide population [1]. Osteoporosis is associated with different health conditions, due to estrogen hormone deficiency during menopause or the consumption of corticosteroids [2]. Additionally, aging, malabsorption of calcium, and immune-related factors can also cause osteoporosis [3]. The bone demineralization processes can lead to fractures, particularly of the vertebrae and hip joint. Several studies indicated that women over 45 years of age are the most vulnerable to femoral fractures [4]. The incidence and cost of these fractures are rising and pose a heavy health and economic burden on the society [5]. Current therapy suffers from various adverse effects; from gastrointestinal disturbances to cancer [6,7]. This necessitates the search for new effective and safe therapies. In this regard, natural products have been advanced as attractive candidates for osteoporosis treatment [3]. Different plant secondary metabolites impact bone remodeling, for example, isoflavonoids (daidzein, genistein, cajanin, and formononetin due to their estrogenic properties), lignans (secoisolariciresinol and matairesinol, which are converted by gut microflora to mammalian lignans, enterodiol and enterolactone that are estrogenic). Furthermore, flavonoids such as quercetin, kaempferol, or hesperidin and carotenoids have all shown preservation of bone mineral density following ovariectomy-induced bone loss in female rodents [8,9].

*Lepidium sativum* L., commercially known as “Hab al Rashad”, grows in Saudi Arabia in several areas, such as AL-Qaseem and the Eastern Province [10]. Traditionally, it is used in folk medicine for bone fractures in the Arabian Peninsula [11]. Phytochemically, the seeds are rich in alkaloids (dimeric imidazole alkaloids lepidine B, C, D, E, and F) [12], anthracene glycosides, saponins, proteins and amino acids, carbohydrates, *S*-glycosides (allyl, 2-phenethyl, and benzyl glucosinolates), and flavonoids [13]. Experimentally, it has been reported to exhibit potent antihypertensive, hypoglycemic, and antioxidant activities [14]. In addition, it has been shown that the seeds are able to accelerate healing of the bone in glucocorticoid-induced osteoporosis in rats [2,15]. Moreover, it was able to accelerate healing of artificial fractures in the midshaft of the left femur of rabbits [11,16]. However, its anti-osteoporotic effects in ovariectomized rats and the possible underlying mechanisms have not been elucidated yet. Further, there is scarcity of data regarding a complete profiling of their active constituents and their possible role in bone healing. This study aimed to evaluate the osteoprotective effects of *L. sativum* seed extract in ovariectomized rats and characterize the metabolite composition of the extract by large-scale chemical profiling using UHPLC/PDA/ESI-MS and GC/MS.

## 2. Material and Methods

### 2.1. Plant Material

*Lepidium sativum* L. seeds (Hab el-Rashad) (Brassicaceae) were purchased from a local market in Jeddah and were identified by Dr. Faraj Alghamdi, Associate Professor of Plant Taxonomy, Biological Science Department, Faculty of Science, King Abdulaziz University. A specimen was kept at the Herbarium of the Faculty of Pharmacy, King Abdulaziz University (LS-1040).

### 2.2. Extraction of Plant Material

One kilogram of *L. sativum* seeds was grounded to a fine powder and extracted at room temperature with methanol (3 × 1 L) till exhaustion. The extracts were refrigerated for future biological examination after evaporation under low pressure.

### 2.3. Biological Study

#### 2.3.1. Determination of Oral Lethal Dose 50 (LD_50_)

LD_50_ was determined in a separate experiment. A limiting dose of 2000 mg/kg of *L. sativum* methanol extract was tried in three female rats and observed for 24 h. As no mortality was observed, the same procedure was repeated using additional three female rats; guided by OECD (Organization for Economic Co-operation and Development) guideline no. 423 [17].

#### 2.3.2. Animals and Animal Treatment

Forty-five female Wistar rats weighing 190–220 g, from the Animal Facility, King Abdulaziz, University, Jeddah, Saudi Arabia were used in the study. They were maintained on a 12-h light–dark cycle and at an ambient temperature of 22 ± 2 °C. Protocols were approved by The Unit of Research Ethics, Faculty of Pharmacy, King Abdulaziz University, Saudi Arabia (PH-118-40). Animals were separated into five groups (9 each) randomly. Animals in group 1 were controls (sham) and were subjected to bilateral laparotomy only, without removing the ovaries. Animals in groups 2, 3, 4, and 5 were subjected to bilateral ovariectomy. All animals were left for 2 weeks to recover from surgery. Rats in group 1 (control) and group 2 were given a daily oral gavage of distilled water (10 mL/kg). Groups 3 and 4 were given an oral gavage of *L. sativum* extract suspended in distilled water at a dose of 50 and 100 mg/kg, respectively. A pilot study was performed to validate the chosen doses, which were also concurrent with the literature [18,19]. Animals in group 5 (positive drug control) were given 17β-estradiol (E2) dissolved in corn oil (25 mcg/kg/day, s.c.) [20,21] for a period of 12 weeks. Individual body weights were recorded at the end of the experiment. Light ether anesthetic was used for collection of blood from the retro-orbital plexus. Blood was left for coagulation (15 min), then centrifuged (2000× *g* for 15 min) and sera were collected and kept at −80 °C. Then, animals were sacrificed under light ether anesthesia by decapitation. Bones of three rats from each group were used for compression strength assessment. For the remaining six rats in each group, right femurs were quickly dissected out, cleaned of soft tissue, weighed, and stored for biomechanical analysis at −80 °C. After dissection and cleaning of the left femurs, they were placed in 10% formalin for future histopathological examination.

#### 2.3.3. Determination of Relative Weights and Compression Strength

Both femurs from each rat (*n* = 3) were completely separated and weighed. The relative bone weights were calculated as follows: wet femur weight/body weight and expressed multiplied by 1000. Femur hardness was determined by a hardness tester (Erweka GmbH, Heusen-stamm, Germany) [22]. Femurs were placed longitudinally (*n* = 3) and perpendicularly (*n* = 3) to the force direction momentarily in the clamp assembly of the tester, and the minimal force causing bone shaft fracture was determined.

#### 2.3.4. Histological Examination

Ten percent neutral buffered formalin was used for fixing the excised femurs followed by dehydration in ethanol, xylene clearing, and embedding in paraffin. On glass slides, cross vertical sections (5 μm) of bone tissues were mounted. Hematoxylin and eosin (H&E), Masson trichrome, and alizarin red stains were applied after dewaxing, and rehydration with distilled water. The samples were examined in a blinded fashion by an expert pathologist. Bone lesions were given scores as follows: Nil, -; Mild, +; Moderate, ++; Severe, +++.

#### 2.3.5. Assessment of Serum Markers of Bone Metabolism

Serum alkaline phosphatase (ALP) activities and osteocalcin levels were determined using rat ELISA kits (Enzyme-Linked Immunosorbent Assay) (Wuhan USCN Life Science, Wuhan, China). A rat ELISA kit (Biomatik, Inc., Ontario, Canada) was used to assay collagen cross-linking carboxyterminal telopeptide, type I (CTXI). Another rat ELISA Kit (MyBioSource, San Diego, CA, USA) was employed for tartrate-resistant acid phosphatase (TRAP) determination.

#### 2.3.6. Preparation of Bone Homogenate

Bones were homogenized (10% of w/v in ice-cooled 0.1 M phosphate buffer, pH 7.4) using a polytron homogenizer. This was followed by centrifugation for 20 min at 4 °C and 10,000× *g*. Oxidative status markers and protein concentration were determined from the supernatant.

#### 2.3.7. Bone Tissue Oxidative Status Assessment

Oxidative status was assessed from tissues of the distal epiphysis of the femur. Commercially available kits (Biodiagnostic, Giza, Egypt) were used to determine malondialdehyde (MDA), superoxide dismutase (SOD), and glutathione peroxidase (GPx) activities. Data were normalized to protein content as determined by BCA (bicinchoninic acid) protein assay kit (Sigma-Aldrich, St. Louis, MO, USA).

#### 2.3.8. *RANKL* and *OPG* Assessment via Quantitative Real-Time Polymerase Chain Reaction (PCR)

Bone tissues of the distal epiphysis of the femur were homogenized in an ultrasonic probe. A nucleic acid extraction kit (NucleoSpin^®^, Macherey-Nagel GmbH & Co. KG, Duerin, Germany) was used for RNA extraction. A dual-wavelength spectrophotometer (Beckman, Spectrophotometer, USA) was utilized for determination of RNA purity (A260/A280 ratio) and concentration. Reverse transcription and real-time PCR was undertaken using SensiFAST™ SYBR^®^ Hi-ROX One-Step Kit; cat. no.: BIO-73005 (Bioline, a Meridian Bioscience company, London, UK). PCR amplification reactions were then performed using the following primers purchased from Qiagen, Valencia, CA, USA: tumor necrosis factor receptor superfamily, member 11b (Tnfsf11), also named as osteoprotegerin (*OPG*) (QT00195125); tumor necrosis factor (ligand) superfamily, member 11 (Tnfrsf11b), also named as receptor activator of nuclear factor kappa-Β ligand (*RANKL*) (QT00177170); and the housekeeping (reference) gene (*GAPDH*) (QT00199633) using StepOne Real-Time PCR System (Applied Biosystems, Foster city, CA, USA). The ratio of target gene to *GAPDH*, with respect to control rats was calculated by the ΔΔCT technique and ratio to control values were used to give the results.

#### 2.3.9. Statistical Analysis

Data are represented as mean ± SD. Multiple comparisons were performed using one-way analysis of variance (ANOVA) followed by Tukey post hoc test. All analyses were performed using IBM SPSS^®^ ver. 25 (SPSS Inc., Chicago, IL, USA). Differences were considered significant at *p* < 0.05.

### 2.4. Metabolites Characterization of *L. sativum* Seed Extract

#### 2.4.1. Extraction and Preparation of Samples for UHPLC/PDA/ESI-MS

*L. sativum* seeds were ground with liquid nitrogen and sixteen mg of the ground material was homogenized using a Turrax mixer with methanol (1.5 mL) containing umbelliferone as an internal standard (10 µg/mL). Prepared extracts were purified from plant debris by centrifugation for half an hour at 3000× *g*. An aliquot of 3 µL of the supernatant was employed in the UHPLC/PDA/ESI-ion trap MS analysis according to the chromatographic conditions and mass spectrometer parameters described previously [23,24].

#### 2.4.2. Analysis of UHPLC-Orbitrap-HRMS

An Orbitrap Elite mass spectrometer (Thermo Fisher Scientific, Darmstadt, Germany) was used for high-resolution ESI-MS in positive and negative modes, and collision-induced dissociation (CID) spectra and conditioning of UHPLC as described previously [25]. Mass spectra, V-VIS spectra (220–600 nm), phytochemical dictionary of natural products database (CRC) literature was used to characterize metabolites.

#### 2.4.3. Volatiles Analysis of *L. sativum* Seeds via Headspace GC/MS

The protocol by Farag et al. (2017b) was used for HS-SPME volatile analysis [26]. Retention indices (RI) were compared to relative to n-alkanes (C6–C20), mass matching to NIST, WILEY library database and with standards for identification of volatile components. Before matching mass spectra, AMDIS software (www.amdis.net) was used for peak deconvolution.

## 3. Results and Discussion

Osteoporosis is caused by different risk factors, including estrogen hormone deficiency after menopause, consumption of corticosteroids, aging, malabsorption of calcium, or immune-related factors. Treatment of osteoporosis by synthetic drugs involves a wide range of side effects. Natural treatments may constitute a healthier and safer treatment option. Herbal remedies have been exploited worldwide due to their efficacy, relative safety, and low-cost. Several plant extracts and natural compounds have been examined for their potential role in protecting against osteoporosis. Many natural active constituents have demonstrated positive effects in bone formation in osteoporosis: including flavonoids (icariin, naringin, ugonin K, genistein, puerarin, quercetin, and rutin), phenyl ethanoids (echinacoside), phenolic acids (salvianolic acid B), diterpenoid (kirenol), sesquiterpene (costunolide), stilbene (kobophenol A), and coumarins (imperatorin and bergapten) [3]. This study was designed to explore the mechanism of osteoprotective activity of *L. sativum* seed extract in ovariectomized rats. Characterization of the extract’s metabolite composition was assessed using large-scale chemical profiling using UHPLC/PDA/ESI–MS and GC/MS.

### 3.1. Biological Study

#### 3.1.1. Oral LD_50_ of *L. sativum* Extract

Twenty-four hours after an oral dosing of 2000 mg/kg of *L. sativum* methanol extract to rats, no deaths were recorded in the three tested female animals. Further studies were carried out in three additional female animals using the same dose, which similarly resulted in no deaths in 24 h. 

*L. sativum* (LD_50_ > 2000 mg/kg) is considered to be Category 5 based on the Acute Toxic Class Method reported in OECD guideline no. 423.

#### 3.1.2. Effect of *L. sativum* on Femur Relative Weight and Compression Strength

**Figure 1:**
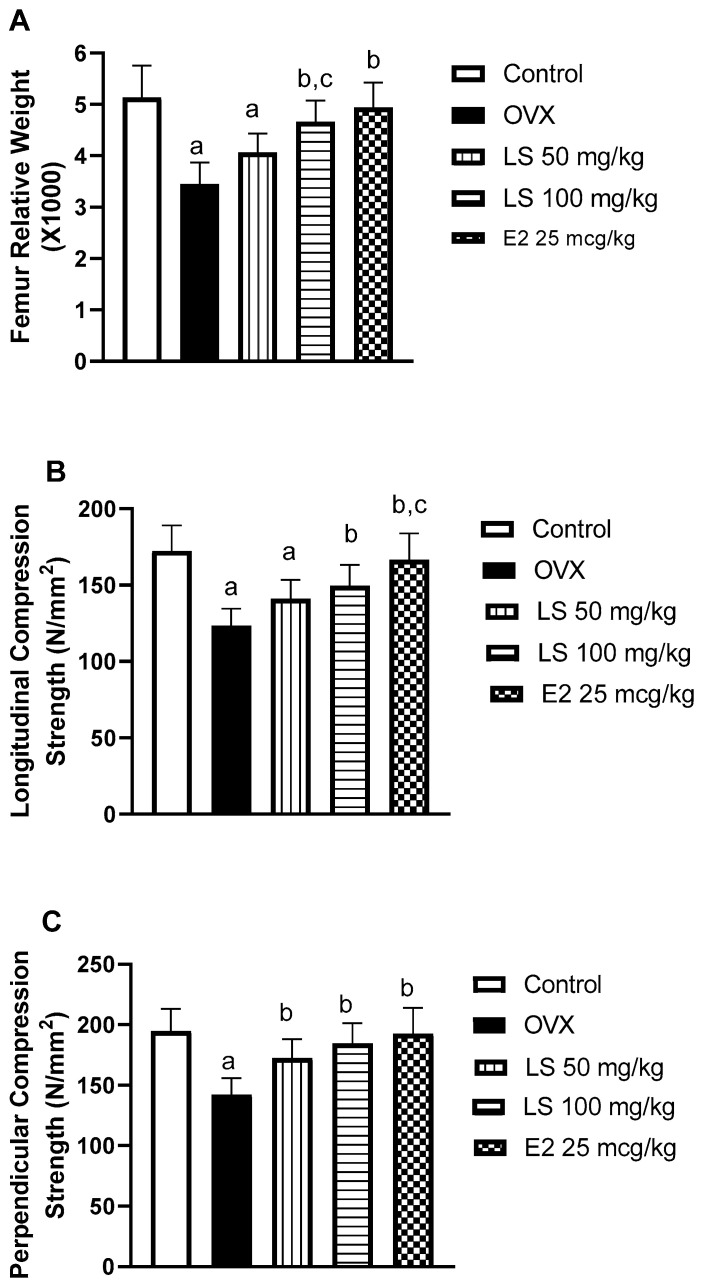
Effect of *Lepidium sativum* (LS) on femur relative weight and compression strength in ovariectomized rats. (**A**) Femur relative weight, (**B**) longitudinal force, (**C**) femur perpendicular force. Data are presented as Mean ± Standard Deviation (M ± SD). (a) Significantly different from control at *p* < 0.05. (b) Significantly different from ovariectomized animals (OVX) at *p* < 0.05. (c) Significantly different from LS 50 mg/kg at *p* < 0.05.

To determine the effects of *L. sativum* on ovariectomy-induced osteoporosis, femur relative weights were determined. Compared with the control (sham) group, ovariectomy (c) resulted in an almost one-third decrease in bone relative weight. The observed osteoporosis induced by ovariectomy is consistent with many previous clinical and experimental reports [27,28]. However, treatment with *L. sativum* resulted in significant enhancement of bone relative weight by 18% and 35% respectively, as compared to the ovariectomized animals (OVX). It is noteworthy to report that *L. sativum*’s effects were comparable to that of the estradiol group (Figure 1A). Further, the anti-osteoporotic activities of *L. sativum* extract are supported by previous reports highlighting its synergistic effects with alendronate against glucocorticoid-induced osteoporosis in rats [2]. To confirm these data, longitudinal and perpendicular femur bone strengths were assessed. Ovariectomy significantly decreased longitudinal and perpendicular compression strengths by 38% and 41% respectively, of the control value. A daily dose of 50 mg/kg of *L. sativum* enhanced the longitudinal and perpendicular strengths by 33% and 52%, respectively. The higher dose (100 mg/kg) resulted in an improvement of both strengths by 49% and 62%, respectively (Figure 1B,C). These observations suggest that *L. sativum* could significantly ameliorate bone-weakening. The observed bone-strengthening activity of *L. sativum* is in line with the clinical notion that the plant improved chronic periodontitis in osteoporotic postmenopausal women as indicated by radiography and clinical signs and symptoms [29].

#### 3.1.3. Histological Examination of Femurs

**Figure 2:**
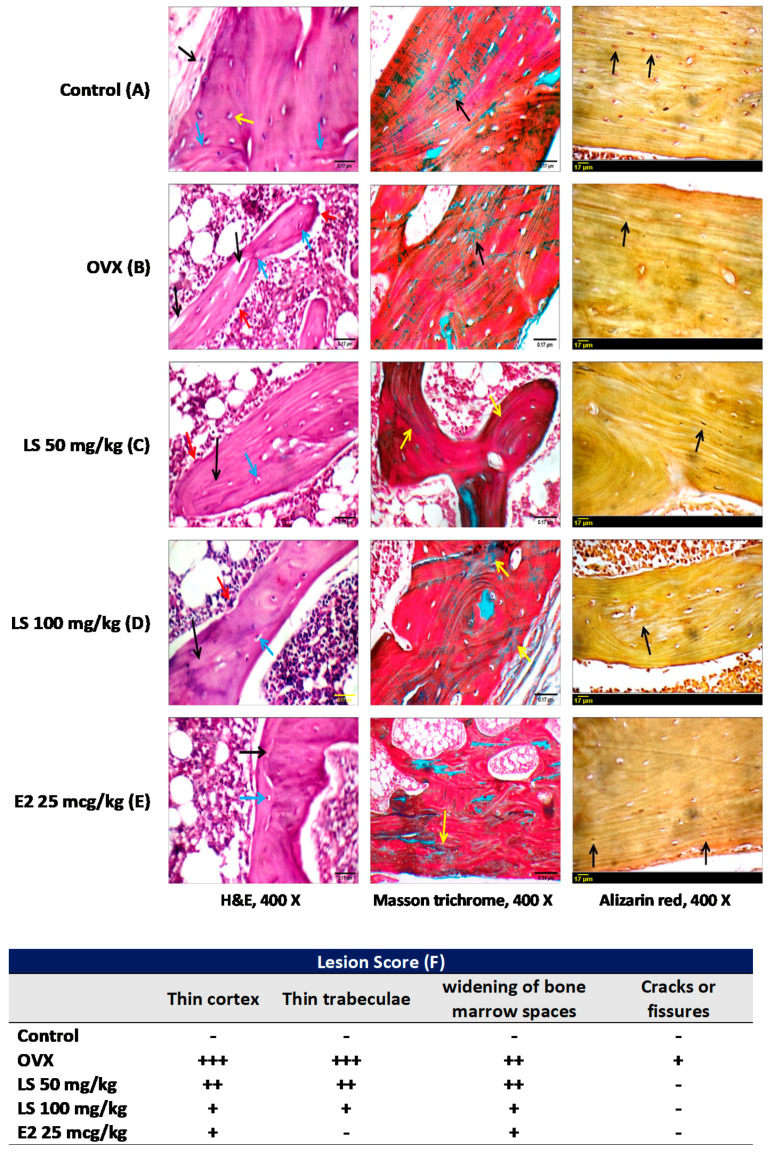
Effect of *L. sativum* on OVX-induced bone matrix and histological alterations using hematoxylin and eosin (H&E), Masson trichrome, and alizarin red stains. (**A**) Control group showing average periosteum (black arrow), average cortex with regular dense lamellae (blue arrows), small osteocytes (yellow arrow) (H&E), cortex with parallel regular collagen fibers (black arrow) (Masson trichrome), cortex reactive for alizarin red with regular lamellae with parallel small osteocytes (black arrow) (alizarin red). (**B**) Ovariectomized untreated animals showing thin eroded bony trabeculae (black arrow) with large osteocytes (blue arrow) and osteoblastic rimming (red arrow) (H&E), irregular arrangement of collagen fibers in cortex (black arrow) (Masson trichrome), non-reactive cortex for alizarin red showing irregular lamellae (black arrow) (alizarin red). (**C**) Ovariectomized rats treated with 50 mg/kg *L. sativum* extract demonstrating bony trabeculae with irregular dense lamellae (black arrow), large osteocytes in large lacunae (blue arrow), and osteoblastic rimming (red arrow) (H&E), regular arrangement of collagen fibers in trabeculae (yellow arrow) (Masson trichrome) and cortex less-reactive for stain (black arrow) (alizarin red). (**D**) Ovariectomized rats treated with 100 mg/kg *L. sativum* extract illustrating bony trabeculae (black arrow), large osteocytes in large lacunae (blue arrow) (H&E), irregular arrangement of dense collagen fibers (yellow arrow) (Masson trichrome), and some reactivity for alizarin (black arrow) (alizarin red). (**E**) Estradiol-treated group displaying average trabeculae with dense lamella (black arrow) small osteocytes in small lacuna (blue arrow) (H&E), irregular arrangement of collagen fibers in cortex (yellow arrow) (Masson trichrome), and frequent parallel small osteocytes reactive to alizarin red (black arrow) (alizarin red). (**F**) Table displaying the lesion scores for the different treatment groups. Nil, -; Mild, +; Moderate, ++; Severe +++.

In addition to classical examination of sections stained with H&E, femur sections were also stained with Masson trichrome and alizarin red stains to give better detection of collagen fibers and calcium-containing osteocytes. Control animals showed average periosteum, normal cortical thickness with regular dense lamellae, intact well-formed dense bony trabeculae with dense lamellae, and average intervening bone marrow. Moreover, Masson trichrome staining showed parallel regular arrangement of collagen fibers. Alizarin red stained-sections exhibited regular cortex and trabeculae (Figure 2A). Animals in the OVX group displayed a thin eroded cortex with large osteocytes in large lacunae, foci of calcified cartilage, thin eroded disconnected and hypo-mineralized bony trabeculae with osteoclastic activity. Masson trichrome-stained sections showed irregular arrangement of collagen fibers while alizarin red staining showed almost non-reactive cortex and trabeculae (Figure 2B). Treatment of ovariectomized animals with *L. sativum* (50 or 100 mg/kg) showed relatively thin cortex with irregular dense lamellae and large osteocytes in large lacuna, well-formed relatively thin eroded bony trabeculae with regular and irregular dense lamellae, osteoblastic rimming with minimal osteoclastic activity, foci of calcified cartilage, regular arrangement of collagen fibers in cortex and trabeculae, and trabeculae almost non-reactive for alizarin red (Figure 2C,D). Animals in the positive control group treated with estradiol showed almost normal femur architecture (Figure 2E). Injury scoring in all treatment groups is shown in a tabular presentation (Figure 2F). The table indicates that ovariectomy resulted in obvious signs of osteoporosis that include thinning of the cortex and trabeculae, widening of bone marrow spaces, and a few fissures. Nevertheless, *L. sativum* in both doses (50 and 100 mg/kg) ameliorated all these pathological effects that are comparable to that of estradiol (Figure 2F).

Histopathological observations reported in this study are concurrent with a recent histological study highlighting the ability of *L. sativum* to protect against glucocorticoid-induced bone histological and morphometric pathological changes [29].

#### 3.1.4. Effect of *L. sativum* on Serum Biomarkers of Bone Formation and Resorption

Serum markers of bone formation ALP activity and osteocalcin level were significantly decreased by ovariectomy by 36% and 25%, respectively as compared to control values (Table 1). Treatment of ovariectomized rats with *L. sativum* ameliorated such pathological alterations. The high dose (100 mg/kg) prevented the decrease of both ALP and osteocalcin by 36% and 29% respectively, as compared to OVX animals. TRAP and CTXI are critical effectors mainly expressed by osteoclasts and play key roles in bone resorption. Therefore, they are excellent indicators of osteoclast activity [30] and their high levels have been linked to bone disorders including osteoporosis. The observed inhibition of their levels suggests a decrease in the intensity of osteoclastogenesis and osteoclast activity. The serum markers of bone resorption TRAP level and CTXI levels were elevated by ovariectomy. *L. sativum* in both doses (50 and 100 mg/kg), as well as estradiol, significantly inhibited rising bone resorption markers TRAP and CTXI. The high dose of *L. sativum* (100 mg/kg) improved ALP and CTXI levels by 28% and 30%, respectively, when compared to animals in the OVX group (Table 1). Thus, the anti-osteoporotic activity of the plant was confirmed by its ability to ameliorate reduced bone formation markers, serum ALP activity, as well as osteocalcin.

#### 3.1.5. Effect of *L. sativum* on Ovariectomy-Induced Oxidative Stress in Bone Tissue

**Figure 3:**
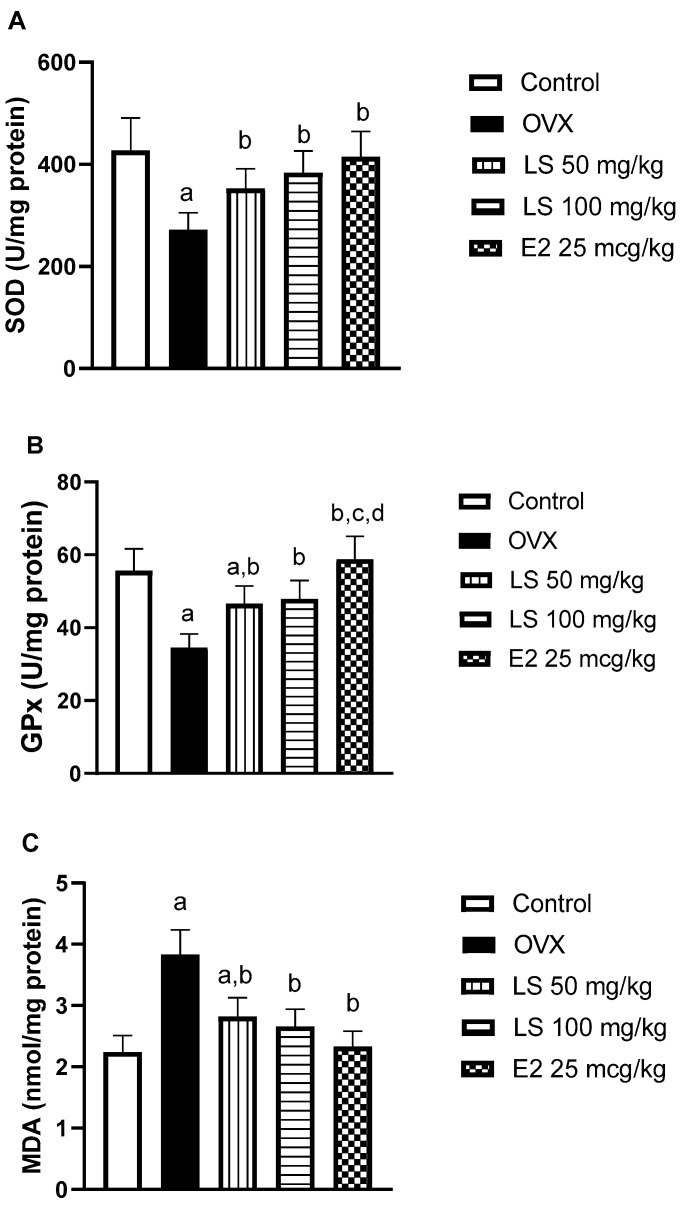
Effect of *L. sativum* (LS) on superoxide dismutase (SOD) activity (**A**), glutathione peroxidase (GPx) activity (**B**) and malondialdehyde (MDA) content (**C**) in femur tissues of ovariectomized rats. Data are presented as M ± SD. (a) Significantly different from control at *p* < 0.05. (b) Significantly different from OVX at *p* < 0.05. (c) Significantly different from LS 50 mg/kg at *p* < 0.05. (d) Significantly different from LS 100 mg/kg at *p* < 0.05.

Ovariectomized animals had reduced antioxidant defense in tissues of the femur with a depletion of SOD and GPx activities by 36% and 38%, respectively, relative to control animals. However, treatment of ovariectomized rats with *L. sativum* significantly prevented the decreased enzymatic activities. In particular, the high dose (100 mg/kg) was able to normalize LDH and GPx values (Figure 3A,B). Assessment of lipid peroxide content in bone tissues, due to oxidative stress, showed high levels in femurs of ovariectomized rats by approximately, 70% in comparison to controls. Treatment of *L. sativum* extract at 50 or 100 mg/kg significantly inhibited the rise in MDA values by 26 or 30, respectively, as compared to OVX values (Figure 3C). Estradiol, as a positive control, could normalize SOD, GPx, and MDA values (Figure 3A–C).

These data are in accordance with many previous studies that reported the anti-oxidant activity of *L. sativum* [19,31,32,33]. There is ample evidence linking oxidative stress to the development of post-menopausal osteoporosis [34,35]. Bone resorption has been shown to occur due to the disturbance in mineral tissue homeostasis resulting from increased reactive oxygen species (ROSs) [36]. ROSs have been recognized as signaling intermediates for osteoclasts, and their effects may be limited by antioxidants due to decreased *in vivo* resorption [37]. The role of antioxidants in preventing bone degeneration has been reviewed [38], to confirm that the observed antioxidant activities of *L. sativum* can be contributed to its anti-osteoporotic activities.

#### 3.1.6. Effect of L. sativum on RANKL and OPG mRNA Expression

**Figure 4:**
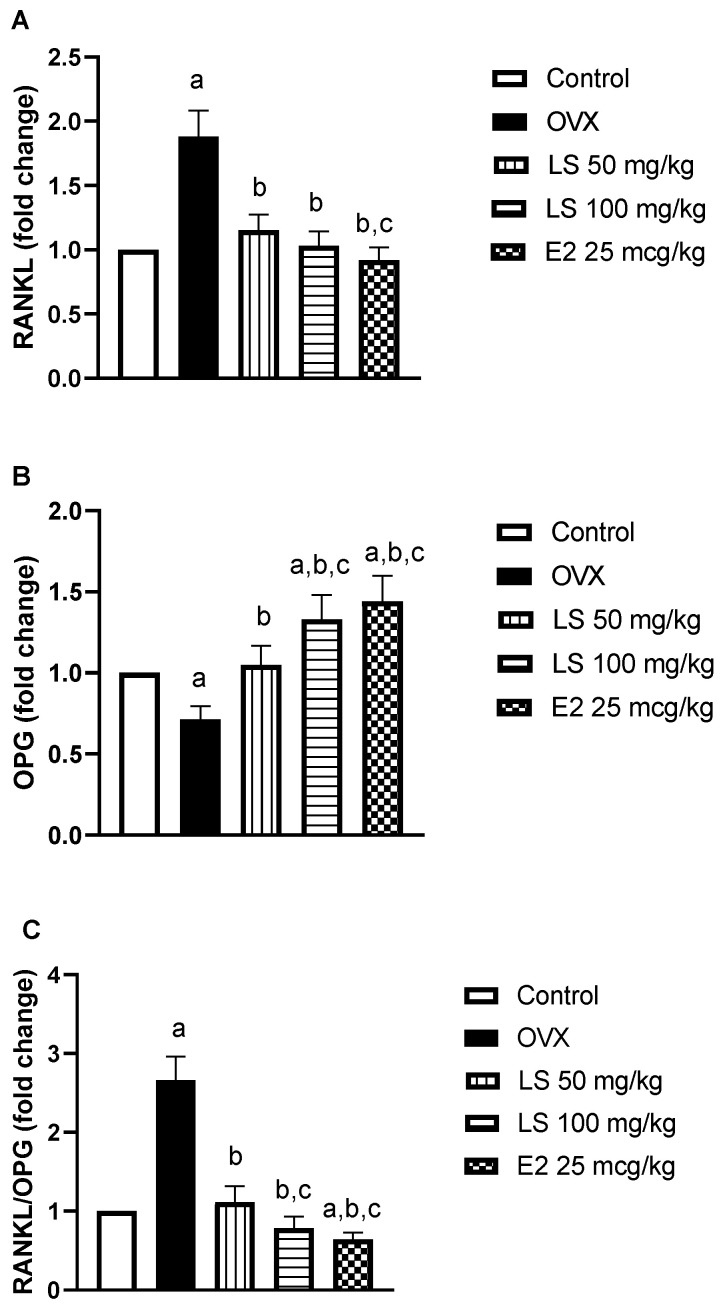
Effect of *L. sativum* (LS) on mRNA expression of receptor activator of nuclear factor kappa-Β ligand (*RANKL*) and osteoprotegerin (*OPG*) and *RANKL/OPG* ratio in femur bones of ovariectomized rats. (**A**) The levels of *RANKL* mRNA transcripts; (**B**) the levels of *OPG* mRNA transcripts; (**C**) *RANKL/OPG* ratio. Data are expressed as means ± SD. (a) Significantly different from control at *p* < 0.05. (b) Significantly different from OVX at *p* < 0.05. (c) Significantly different from LS 50 mg/kg at *p* < 0.05.

*RANKL* mRNA was significantly upregulated in the femur bones of ovariectomized rats by almost 9-fold relative to controls. This was accompanied by a 29% reduction in *OPG* expression in comparison to controls. The *RANKL*/*OPG* ratio rose significantly by approximately 1.6-fold. Nevertheless, treatment of rats with *L. sativum* (50 or 100 mg/kg) significantly reduced ovariectomy-induced *RANKL* expression by 38% and 45% respectively, as compared to ovariectomized animals. In addition, *L. sativum* (50 or 100 mg/kg) significantly enhanced mRNA expression of *OPG* by 47% or 80%, respectively, as compared to ovariectomized rats. Estradiol normalized *RANKL* expression, enhanced *OPG* expression and significantly decreased the *RANKL/OPG* ratio as compared to control animals (Figure 4A–C).

The detected *RANKL* upregulation, a marker of osteoclastic activity, in ovariectomized rats may have occurred as a result of oxidative stress. Oxidative stress and ROS have been previously associated with *RANKL* upregulation [39]. Conversely, antioxidants protected against H_2_O_2_-mediated upregulation of *RANKL* [40]. This observation is consistent with the enhancement of the mRNA expression in the bone formation marker *OPG*. Fundamentally, *OPG*, also known as osteoclastogenesis inhibitory factor (OCIF), is essential for bone remodeling, a normal process in which old bone is broken down and new bone is created to replace it. Further, *L. sativum* prevented the rise of *RANKL/OPG* ratio, which is an essential component in the regulation of bone metabolism [41]. Menopause has been reported to disrupt the *RANKL/OPG* system and enhance risk for osteoporosis [42]. In addition, the potential estrogenic activity of *L. sativum* cannot be excluded. Supplementation of *L. sativum* seed was reported to stimulate gonadotropin secretion in rabbits. This was attributed to its rich phytosterol content that activates estrogen receptors producing agonistic effects [43]. Furthermore, the volatile oil obtained by steam distillation of *L. sativum* was shown to exhibit an estrogenic effect accompanied by an increase in the weight of ovariectomized rats [44]. Thus, the estrogenic activity of the extract could have an important role in modulating *RANKL/OPG* mRNA expression and mediate the anti-osteoporotic activities of *L. sativum*. Determination of the estrogenic effect of *L. sativum* extract in ovariectomized rats should help prove such hypothesis which is yet to be determined.

### 3.2. Metabolites Characterization of *L. sativum *Seed Extract via UPLC and GC/MS Analyses

**Figure 5:**
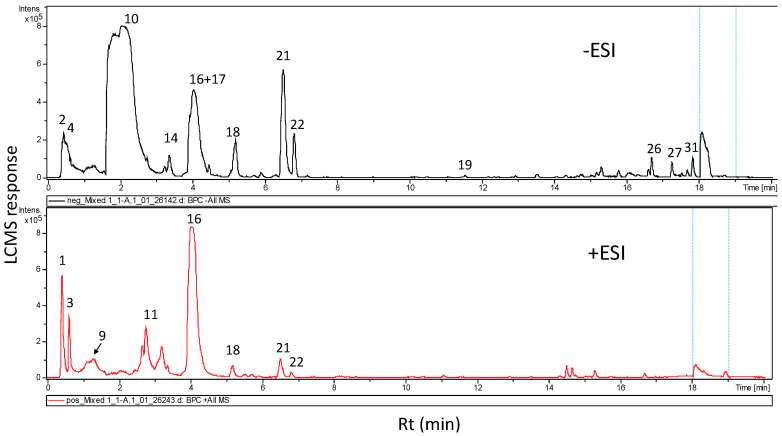
Representative UHPLC-MS base peak chromatogram of *L. sativum* 100% methanol seed extract in negative mode (black color) and positive (red color) ionization mode. Peak numbers follow those listed in Table 2. (Rt.: retention time).

High-resolution UHPLC-PDA-ESI-MS was used for analysis of *L. sativum* seed methanolic extract using positive and negative ionization (Figure 5). Gradient elution of seed metabolites resulted in complete elution in 21 min and gave a complete profile of this genus’s seed metabolome from Saudi Arabia. UPLC-MS has been employed for profiling of *L. sativum* sprouts leading to the identification of sinapine and acylated flavonoids as major constituents [45]. Representative UHPLC-MS traces of seed 100% methanolic extract in (+/−) ESI modes are given (Figure 5). The two ionization modes have similar peak responses, except for glucosinolate peak 10, which showed preferential ionization in negative mode versus abundance of alkaloid i.e., peaks 3 and 16 in the early elution portion of the chromatogram (0–4 min) as detected in positive ionization mode. A stronger response was observed in negative modes for fatty acids showing late elution, and as expectedly detected between rt (retention times)16 and 18 min. MS data (accurate molecular ion mass and fragmentation pattern), UV spectra, and elution times were factors in determining metabolite assignment relative to reports on *Lepidium* metabolites as shown in (Table 2). Classes of metabolites that were identified include flavonoids, alkaloids, glycoalkaloids, glucosinolates, phenolic/fatty acids, and coumarins with alkaloids and glucosinolates as the most abundant classes.

#### 3.2.1. Alkaloids

Alkaloids eluting in the earlier elution region of the chromatogram were detected almost exclusively in the positive ionization mode due to presence of nitrogen atoms. The identified alkaloids included a glycoalkaloid peak 3 rt 0.5 min ([M + H]^+^ 337.1384, C_16_H_21_N_2_O_6_) and aglycone in peaks rt 2.9 min ([M + H]^+^ 347.1204, C_20_H_19_N_4_O_2_) and rt 3.7 min ([M + H]^+^ 361.167, C_21_H_21_N_4_O_2_). Peak 3 showed a loss of 30 amu and 162 amu in respective fragment ions at *m/z* 347 and *m/z* 174, indicative of methoxy and hexosyl moieties and confirming its annotation as semilepidinoside A. Aglycone peaks 13 and 14 showing later elution were annotated as lepdine B/D/E/F and lepidine C, respectively, all belong to the imidazole alkaloid class type typical of *L. sativum* [12]. A fragment ion appearing at *m/z* 174 was detected in MS fragmentation of imidazole alkaloids and its glycoside and served in its identification. Sinapine was present as the major alkaloid detected in peak 16 [M + H]^+^ at *m/z* 310.1657 and displaying characteristic fragment ions at *m/z* 251 and 207 [45], and previously reported in *L. sativum* sprouts.

#### 3.2.2. Glucosinolates

Benzyl glucosinolate (glucotropaeolin) was identified as major glucosinolate in *L. sativum* (peak 10, *m/z* 408.0463, C_14_H_19_NO_9_S_2_^−^) and showing loss of 162 amu, previously reported in in LC/MS analysis of *Lepidium meyenii* [46,47]. The cytotoxic and antimicrobial properties of *Lepidium latifolium* hydrodistillate are mainly due to the presence of glucosinolates [47]. In particular, isothiocyanate, which is a main degradation sulfur volatile allyl is thought to be involved in these activities. Whether allyl isothiocyanate is also present in *L. sativum* has yet to be reported. Analysis of sulfur aroma compounds via GC/MS analysis can provide a complete profile of glucosinolate hydrolysis products [48].

#### 3.2.3. Phenolic Acids & Flavonoids/Lignans

Major contributions to the antioxidant effects of food materials are due to the presence of phenolic acids. They are present in free or conjugated forms, such as flavonoids, and are a common class of secondary metabolites [25]. In this study, three phenolic acid conjugates were detected exclusively in negative ion mode (Table 1) in peaks (4, 18, and 19) as monomer or dimer of methylated caffeic, ferulic, and dimethoxy cinnamic acids.

Few flavonoids of the flavanone subclass and one lignan peak were identified in peaks 1, 5, 11, 15, and 23. Lariciresinol (peak 11) was identified from its characteristic UV absorption at 280 nm and molecular ion [M - H^−^] at *m/z* 359.1525 and displaying fragment ions at *m/z* 345 and 298. Lariciresinol 4′-*O*-*β*-d-glucopyranoside was previously obtained from *L. apetalum* seeds [49].

#### 3.2.4. Coumarins

Two coumarins of esculin aglycone (hydroxycoumarin) were annotated in *L. sativum* as esculin-*O*-hexoside (peak 21 [M - H^−^] at *m/z* 339.0722) and showing loss of 162 amu and dimethylesculin (peak 20 [M - H^−^] at *m/z* 207.0642), and both displaying UV max at 330 nm typical of coumarin. Esculin was previously reported in *Lepidium* in a response to umbelliferone uptake by roots [50].

#### 3.2.5. Fatty Acids

Several unsaturated fatty acids were identified after negative ionization MS, during the final part of the chromatographic run (16–18 min). Negative ion MS spectra of the unsaturated fatty acids: (linolenic acid, peak 26), (linoleic acid, peak 27), (oleic acid, peak 31), (eicosenoic acid, peak 32) were interpreted using exact masses of 277.2173, 279.233, 281.2488, and 311.2941 and an expected molecular formula of C_18_H_29_O_2_^−^, C_18_H_31_O_2_^−^, C_18_H_33_O_2_^−,^ and C_20_H_39_O_2_^−^, respectively. *L. sativum* seed oil showed anti-inflammatory, anti-oxidant, and antimicrobial activities [51], attributed to the presence of unsaturated fatty acids in *L. sativum* seed, which potentially can serve as functional food for hyperlipidemia. Negative ionization MS also revealed the presence of a few hydroxy fatty acids that eluted in peaks 24, 25, and 29. Peak number 25 was identified as hydroxy hexadecanoic acid [M − H]^−^ *m/z* 271.2299, showing fragment ions at *m/z* 225 suggestive for the respective loss of CH_2_O_2_ [52]. This is an unprecedented report for hydroxyl fatty acids in *L. sativum* and is of increasing interest owing to its reported antimicrobial, cytotoxic, and anti-inflammatory actions [53]. Whether these oxylipids contribute to *Lepidium* seed’s reported anti-inflammatory and anti-microbial properties [51] has yet to be confirmed.

#### 3.2.6. Volatile Compounds

**Figure 6:**
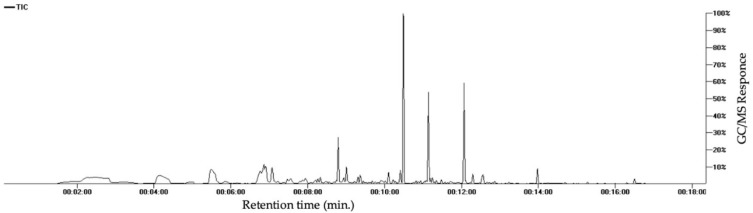
Representative SPME-GC-MS chromatogram of *L. sativum*.

Volatile constituents of the plant were assessed utilizing headspace solid-phase microextraction (SPME) and GC/MS (Figure 6). The results (Table 3) showed enrichment of sesquiterpene hydrocarbons (35.9%), followed by aromatic volatile constituents (20.7%), volatile alcohols (20.6%), volatile acids (7.06%), and aliphatic hydrocarbons (4.82%) in *L. sativum* aroma blend. α-Copaene represented the major sesquiterpene hydrocarbons (22.7%), whereas 1-hexanol was the major alcohol (12.2%).

Based on the analysis performed in this study, it could be concluded that the osteoprotective effect of *L. sativum* is likely due to a synergistic effect of its various classes of metabolites i.e., alkaloids, lignans, phenolic acids, etc. For example, glucosinolates (peak 10) stimulated bone formation in young rats and in MG-63 cells [54]. Phenolic acids were also investigated for their osteoprotective effect in ovariectomized rats, with ferulic, caffeic, *p*-coumaric, and chlorogenic acids to counteract skeletal changes caused by estrogen deficiency in ovariectomized rats [55]. Plant lignans have been shown to provide estrogenic support and antioxidant and anti-inflammatory activity. Lariciresinol, a lignan precursor detected in peak 11, increases bone formation and significantly inhibits resorption.

Identified coumarin, esculin (peak 21) is reported for its osteoprotective effect mediated via modulation of bone metabolism by reducing osteoclastogenesis caused by receptor activator of NF-κB ligand (*RANKL*) and transduction signals in an osteoporosis model of ovariectomized rats [56]. Animal models have suggested that fatty acids as linolenic acid (omega-3), a major oxylipid in *L. sativum* (peak 26) could reduce postmenopausal bone loss. A significantly lower amount of bone was lost at the lumbar vertebrae and femurs of ovariectomized mice receiving a high-omega-3 diet [57]. In addition, it was observed in a clinical study that osteocalcin and procollagen were augmented in the linolenic acid supplemented groups [58]. Thus, *L. sativum* has been suggested for the management of postmenopausal osteoporosis and prevention of estrogen deficiency-induced fractures [59].

## 4. Conclusions

*L. sativum* ameliorates ovariectomy-induced bone injury in rats. This is evidenced by increasing bone weight, enhancing bone formation biomarkers (LDH and osteocalcin), as well as its free radicle scavenging activity of the extract. Moreover, it ameliorates the rise in the markers of bone resorption carboxyterminal telopeptide, type I (CTXI) and tartrate-resistant acid phosphatase (TRAP) and modulates *RANKL/OPG* expression. The synergy between the various constituents present in *L. sativum* most likely plays a contributing role in the osteoprotective effects of *L. sativum*. The current data not only support the folk use of *L. sativum* as a bone healer but also reveal the nature of bio-actives responsible for the observed activity and extraction action mechanism. In addition, our findings warrant further experimental and clinical studies. Whether the improvement in osteoporosis is mediated via an estrogenic effect for *L. sativum* extract has yet to be determined. These results are just a hint and need to be followed by testing isolated compounds using the same bioassay.

## Figures and Tables

**Table 1:** Effect of L. sativum on serum markers of bone formation and resorption.

	ALP (U/dL)	Osteocalcin (pg/mL)	TRAP (U/dL)	CTXI (ng/mL)
**Control**	28.65 ± 2.71	4.25 ± 0.45	2.25 ± 0.32	3.25 ± 0.47
**OVX**	21.33 ± 2.83 ^a^	2.68 ± 0.28 ^a^	4.10 ± 0.45 ^a^	5.47 ± 0.58 ^a^
**LS 50 mg/kg**	25.74 ± 2.88	3.15 ± 0.32 ^a^	3.27 ± 0.34 ^a^	4.10 ± 0.47 ^a,b^
**LS 100 mg/kg**	27.61 ± 2.80 ^b^	3.66 ± 0.38 ^b^	2.94 ± 0.25 ^a,b^	3.85 ± 0.40 ^b^
**E2 25 mcg/kg**	28.47 ± 2.90 ^b^	4.10 ± 0.43 ^b,c^	2.44 ± 0.32 ^b,c^	3.55 ± 0.42 ^b^

Data are presented as M ± SD. ^a^ Significantly different from control at *p* < 0.05. ^b^ Significantly different from OVX at *p* < 0.05. ^c^ Significantly different from LS 50 mg/kg at *p* < 0.05. E2: estradiol, LS: *L. sativum,* ALP: alkaline phosphatase, TRAP: tartrate-resistant acid phosphatase, CTXI: carboxyterminal telopeptide, type I.

**Table 2:** Metabolites identified in 100% methanol extracts of *L. sativum* seeds via UHPLC-PDA-ESI-MS in both negative and positive ionization modes. Nd = not detected, + or – denotes molecular ion charge in molecular formula.

Peak No	RT (min)	UV (nm)	Mol. Ion	Mol. Formula	Name	Class	Error (ppm)	MSMS Fragments
1	0.3	Nd	705.1815	C_36_H_35_O_15_^+^	Hexahydroxy-biflavanone-*O*-hexoside	Flavonoid	−0.1	543
2	0.35	Nd	325.1135	C_12_H_21_O_10_^+^	Unknown disaccharide	Sugar	2.3	223, 127
3	0.5	280	337.1384	C_16_H21N_2_O_6_^+^	Semilepidinoside A	Glycoalkaloid	0.9	347, 174
4	0.52	Nd	719.2016	C_37_H_37_O_15_^+^	Dimethoxycinnamoyl-*O*-feruloyl-O-caffeoylquinic acid	Phenolic acid	−5.4	539, 377, 341
5	0.5	Nd	723.1882	C_36_H_37_O_16_^+^	Tetrahydroxyflavan-tetrahydroxyflavanone hexoside	Flavonoid	4.9	
6	0.57	Nd	539. 1378	C_24_H_27_O_14_^−^	Unknown	Sugar	1.4	377
7	0.6	280	337.1384	C_16_H_21_N_2_O_6_^+^	Semilepidinoside A (isomer)	Glycoalkaloid	2.9	174
8	0.7	Nd	337.1409	C_12_H_17_O_8_^+^	Aconitic acid-*O*-hexoside	Organic acid	7.7	175
9	1.1	280	347.1476	C_20_H_19_N_4_O_2_^+^	Lepidine (B/D/E/F)	Alkaloid	7.4	174
10	1.9	280	408.0463	C_14_H_19_NO_9_S_2_^−^	Benzyl-glucosinolate (Glucotropaeolin)	Glucosinolate	-	246, 212, 96
11	2.3	280	359.1525	C_20_H_23_O_6_^−^	Lariciresinol	Lignan	3.1	345, 298
12	2.6	280	361.1648	C_21_H_21_N_4_O_2_^+^	Lepidine C	Alkaloid	3	174
13	2.9	Nd	347.167	C_20_H_19_N_4_O_2_^+^	Lepidine (B/D/E/F)	Alkaloid		
14	3.2	280	361.167	C_21_H_21_N_4_O_2_^+^	Lepidine C	Alkaloid	3.0	214
15	3.4	280.5	425.0897	C_22_H_17_O_9_^−^	Unknown	Flavonoid	−4.5	359
16	3.9	328	310.1657	C_16_H_25_NO_5_^+^	Sinapine	Alkaloid	0.6	251, 207
17	4.1	328	294.1348	-	Unknown	Glucosinolate		96
18	5.0	Nd	369.1191	C_17_H_21_O_9_^+^	O-Caffeoylquinic acid methyl ester	Phenolic acid	−2.5	
19	5.8	Nd	225.0745	C_11_H_13_O_5_^+^	Trihydroxycinnamic acid-O-di-methyl ester	Phenolic acid	5.3	175
20	6.1	328	207.0642	C_11_H_9_O_4_^−^	Dimethylesculetin	Coumarin	0.4	175
21	6.5	329	339.0722	C_15_H_15_O_9_	Esculin	Coumarin	−4.6	223, 179
22	6.9	327	613.1870	C_26_H_33_N_2_O_15_^+^	Unknown			
23	11.6	326	591.1684	C_28_H_31_O_14_^−^	Acacetin-*O*-rutinoside	Flavonoid	5.9	283
24	12.2	Nd	329.2319	C_18_H_33_O_5_^−^	Trihydroxy octadecenoic acid	Hydroxy fatty acid	4.4	283
25	16.5	Nd	271.2299	C_16_H_31_O_3_^−^	Hydroxy hexadecanoic acid	Hydroxy fatty acid	3.5	225, 116
26	16.6	Nd	277.2173	C_18_H_29_O_2_^−^	Linolenic acid	Unsaturated fatty acid	4.5	251, 211
27	17.2	Nd	279.2330	C_18_H_31_O_2-_	Linoleic acid	Unsaturated fatty acid	4	211
28	17.3	Nd	425.287+	C_24_H_41_O_6_^+^	Unknown steroid	Steroid	3.3	
29	17.5	Nd	327.2888	C_20_H_39_O_3_^−^	Hydroxy eicosanoic acid	Hydroxy fatty acid	5.1	-
30	17.7	Nd	353.3044	C_22_H_41_O_3_^−^	Oxo docosanoic acid	Oxygenated fatty acid	4.9	-
31	17.8	Nd	281.2488	C_18_H_33_O_2_^−^	Oleic acid	Unsaturated fatty acid	−0.7	
32	18.8	Nd	311.2941	C_20_H_39_O_2_^+^	Eicosenoic acid	Unsaturated fatty acid	1.2	

**Table 3:** Relative percentage of components in *L. sativum* seed analyzed using solid-phase microextraction (SPME)-GC-MS, *n* = 3.

No.	CAS no.	Name	Class	RT	KI	Expected KI	%
1	64-19-7	Acetic acid	Acid	2.811	591	610	1.95
2	111-27-3	1-Hexanol	Alcohol	5.473	843	868	12.21
3	142-62-1	Caproic acid	Acid	6.8486	973	990	1.70
4	72237-36-6	4-Hexenyl acetate	Ester	7.2139	1008	1020	0.69
5	535-77-3	*m*-Cymene	Monoterpene hydrocarbon	7.4558	1030	1023	1.29
6	100-51-6	Benzyl alcohol	Aromatic	7.5425	1038	1036	1.21
7	111-87-5	1-Octanol	Alcohol	7.9197	1073	1071	1.41
8	30086-02-3	3,5-Octadien-2-one	Ketone	7.9493	1076	1073	0.84
9	99-86-5	α-Terpinene	Monoterpene hydrocarbon	8.1916	1099	1017	0.59
10	1120-21-4	Undecane	Aliphatic hydrocarbon	8.2512	1104	1100	0.99
11	124-19-6	Nonanal	Aldehyde	8.3168	1110	1104	1.21
14	140-29-4	Benzyl cyanide	Nitrogenous	8.7354	1150	1144	0.97
16	124-07-2	Caprylic acid	Acid	8.924	1167	1180	0.91
17	143-08-8	1-Nonanol	Alcohol	8.9942	1174	1173	3.78
19	91-20-3	Naphthalene	Aromatic	9.2767	1200	1182	0.21
20	112-40-3	Dodecane	Aliphatic hydrocarbon	9.2915	1202	1200	1.21
21	140-67-0	Estragole	Ether	9.3507	1208	1196	1.92
22	112-31-2	Decanal	Aldehyde	9.3689	1210	1206	0.85
23	17301-23-4	2,6-Dimethylundecane	Aliphatic hydrocarbon	9.4331	1217	1210	0.41
28	112-05-0	Nonanoic acid	Acid	9.8915	1265	1273	0.59
30	14371-10-9	Cinnamaldehyde	Aldehyde	10.0882	1286	1270	2.65
31	1197-15-5	Alpha-Terpinen-7-al	Aldehyde	10.2019	1298	1283	0.45
32	629-50-5	Tridecane	Aliphatic hydrocarbon	10.2611	1304	1300	0.29
33	104-54-1	Cinnamyl alcohol	Alcohol	10.3897	1318	1312	3.23
36	80-26-2	α-Terpinyl acetate	Ester	10.8007	1361	1350	0.49
37	104-61-0	γ-Nonalactone	Lactone	10.9187	1373	1363	0.42
38	622-78-6	Benzyl Isothiocyanate	Nitrogenous	10.9927	1381	1364	0.15
39	77-68-9	1,3-Pentanediol, 2, 2,4-trimethyl-, 1-isobutyrate	Ester	11.0303	1385	1380	0.48
40	22469-52-9	Cyclosativene	Sesquiterpene hydrocarbon	11.065	1389	1368	0.46
41	3856-25-5	Alpha-Copaene	Sesquiterpene hydrocarbon	11.1217	1395	1376	22.70
42	629-59-4	Tetradecane	Aliphatic hydrocarbon	11.1699	1400	1400	1.00
43	3650-28-0	Sativene	Sesquiterpene hydrocarbon	11.3348	1416	1396	0.53
44	475-20-7	Longifolene	Sesquiterpene hydrocarbon	11.4616	1428	1405	0.85
45	87-44-5	Caryophyllene	Sesquiterpene hydrocarbon	11.5517	1437	1419	0.13
46	91-64-5	Coumarin	Lactone	11.6997	1451	1441	0.41
47	-	Unknown 6	Aromatic	12.0477	1485	-	16.60
48	629-62-9	Pentadecane	Aliphatic hydrocarbon	12.0883	1489	1500	0.56
49	10208-80-7	α-Muurolene	Sesquiterpene hydrocarbon	12.2757	1507	1440	2.82
50	39029-41-9	g-Cadinene	Sesquiterpene hydrocarbon	12.4499	1524	1513	0.08
51	483-76-1	δ-Cadinene	Sesquiterpene hydrocarbon	12.518	1531	1524	2.12
52	483-77-2	Calamenene	Sesquiterpene hydrocarbon	12.5412	1533	1523	2.23
53	29837-12-5	Cubenene	Sesquiterpene hydrocarbon	12.6469	1543	1532	0.25
54	21391-99-1	α-Calacorene	Sesquiterpene hydrocarbon	12.7865	1557	1542	0.18
55	629-73-2	Cetene	Sesquiterpene hydrocarbon	13.1248	1590	1592	0.09
56	544-76-3	Hexadecane	Aliphatic hydrocarbon	13.2144	1598	1600	0.35
57	119-61-9	Benzophenone	Ketone	13.7904	1662	1635	0.09
59	-	Unknown 8	Unknown	13.9587	1680	-	3.49
60	544-63-8	Myristic acid	Acid	14.6776	1759	1768	0.19
61	120-51-4	Benzyl Benzoate	Ester	14.8996	1784	1762	0.05
63	1002-84-2	Pentadecanoic acid	Acid	15.5098	1848	1867	0.10
65	373-49-9	Palmitoleic acid	Acid	16.2828	1929	1951	0.12
66	57-10-3	Palmitic acid	Acid	16.4798	1949	1968	1.51
							100.00
